# Malalignment and malposition of quadriceps-sparing approach in primary total knee arthroplasty: a systematic review and meta-analysis

**DOI:** 10.1186/s13018-017-0627-7

**Published:** 2017-09-06

**Authors:** Fu-Zhen Yuan, Shao-Jie Wang, Zhu-Xing Zhou, Jia-Kuo Yu, Dong Jiang

**Affiliations:** Institute of Sports Medicine, Peking University Third Hospital, No. 49 North, Garden Road, Haidian, Beijing 100191 China

**Keywords:** Knee arthroplasty, Meta-analysis, Minimally invasive, Quadriceps-sparing, Medial parapatellar

## Abstract

**Background:**

Quadriceps-sparing (QS) approach is considered to be the most minimally invasive surgery for total knee arthroplasty (TKA). We perform this meta-analysis to evaluate whether malalignment and malposition are more biased towards the QS approach compared to the traditional medial parapatellar (MP) approach, which is still controversial.

**Methods:**

According to the PRISMA guidelines, a comprehensive search was conducted in the databases of PubMed, the Cochrane library, and Embase. Relevant measures were extracted independently by two investigators.

**Results:**

Five randomized controlled trials (RCTs) and eight retrospective studies including a total of 1261 cases were identified. The QS approach was associated with more outliers of hip-knee-ankle (HKA) angle (*p* = 0.03), coronal tibial component angle (*p* = 0.03), and femoral notch (*p* = 0.05). However, the differences of the outlier of the coronal femoral component angle between the two groups were not statistically significant.

**Conclusions:**

This meta-analysis indicates that the QS approach is related to the high risk of malalignment and malposition. However, different studies reported different indicators resulting in small samples for analyzing the radiological outcomes. In addition, both of the relatively long learning curve and the present instruments might increase the risk of malalignment and malposition of the QS approach, which needs further study and improvement.

## Background

Total knee arthroplasty has been a very successful treatment for alleviating pain and restoring physical function in patients with advanced arthritis of the knee [1–4]. In recent years, many reports have focused mainly on minimally invasive surgery (MIS) total knee arthroplasty (TKA) which includes mini-medial parapatellar, midvastus (MV), subvastus (SV), and quadriceps-sparing (QS) approaches in TKA. The goal of the MIS TKA was to decrease the pain with faster recovery via less soft tissue injury, and the QS approach is considered the least invasive about violating the quadriceps muscle [5]. Critics have raised questions about component positioning and limb malalignment [6, 7] while the originators have claimed that no compromises occur with respect to limb malalignment or to the soft tissue about the knee [5, 8, 9]. The excellent mechanical axis of the limb has been proven to be associated with a better outcome [10, 11], and the correct component position has been implicated as a significant factor affecting the longevity of prosthesis [10–13]. Incorrect positioning of the implant and improper alignment of the limb can lead to accelerate implant wear and loosening, as well as suboptimal function [14, 15]. So we performed this meta-analysis to confirm whether traditional medial parapatellar (MP) approach had superiority in limb alignment and positioning of prosthesis. Traditional MP TKA is a medial parapatellar arthrotomy with a larger incision, eversion of the patellar, and full tibia anterior dislocation [16] and QS TKA is a technique avoiding injuring the quadriceps without patellar eversion or tibia anterior dislocation [5].

## Methods

### Search strategy

A detailed search of the following databases of all relevant literature according to the Cochrane Handbook [17] was performed within the period 2006 to March 2017: PubMed, Embase, and Cochrane Collaboration Library. There were no restrictions on language, publication type, and region. And search strategies were used with different combinations of the following keywords: (knee arthroplasty OR knee replacement) AND (quadriceps-sparing OR quadriceps sparing OR quad-sparing OR quad sparing OR minimally invasive OR mini-incision). In order to avoid omitting relevant clinical trials, we scanned the reference lists of articles identified in the initial searches and conference summaries.

### Inclusion and exclusion criteria

Eligible studies were evaluated independently by two investigators (Yuan FZ and Wang SJ). Only those meeting the following criteria were selected for subsequent analysis:

1. Studies comparing the limb alignment and prosthesis position outcomes in MP and QS approaches in TKA
2. Study design: randomized controlled trials and retrospective comparative studies (both cohort and case-control studies)
3. Study population: patients with knee arthritis undergoing primary TKA
4. Intervention: including both QS TKA and MP TKA

Fracture deformity, tumor, animal and cadaver studies, review articles, case report, editorials, and letters to the editor were excluded.

### Data extraction

Two reviewers independently checked all potentially suitable studies using a pre-designed sheet to perform data extraction. Any disagreements were resolved by discussion. Extracted data included first author, publication year, country, sample size, mean duration of follow-up, prosthesis type, and the matching of sex, age, body mass index (BMI), follow-up, preoperative visual analog scale (VAS), preoperative range of motion (ROM), and preoperative deformity. If outcomes were presented from studies at different time points, we extracted data from the latest postoperative time point. For data that could not be directly obtained, we dispatched e-mails to the author and researched other studies citing the trial in question.

### Methodological quality assessment

The quality of those included RCTs was assessed independently using the Cochrane Handbook for Systematic Reviews of Interventions [18], and the quality of cohort studies and case-control studies was evaluated by Newcastle-Ottawa Scale (NOS) [19] which is a simple tool and has been recommended by Cochrane collaboration [17].

### Statistical analysis

Review Manager 5.3 (Cochrane Collaboration, Oxford, UK) was used for statistical analysis. For dichotomous variables, odds ratio (OR) and 95% confidence intervals (CIs) were calculated and graphical output was documented by forest plots. A funnel plot was constructed to assess publication bias for the primary outlier of hip-knee-ankle (HKA) angle. Statistical heterogeneity was evaluated with the *I* ^2^ statistic and the chi-squared (*χ* ^2^) test. A *P* > 0.1 and an *I* ^2^ ≤ 50% were considered no or low statistical heterogeneity.

## Results

### Search results

**Fig. 1:**
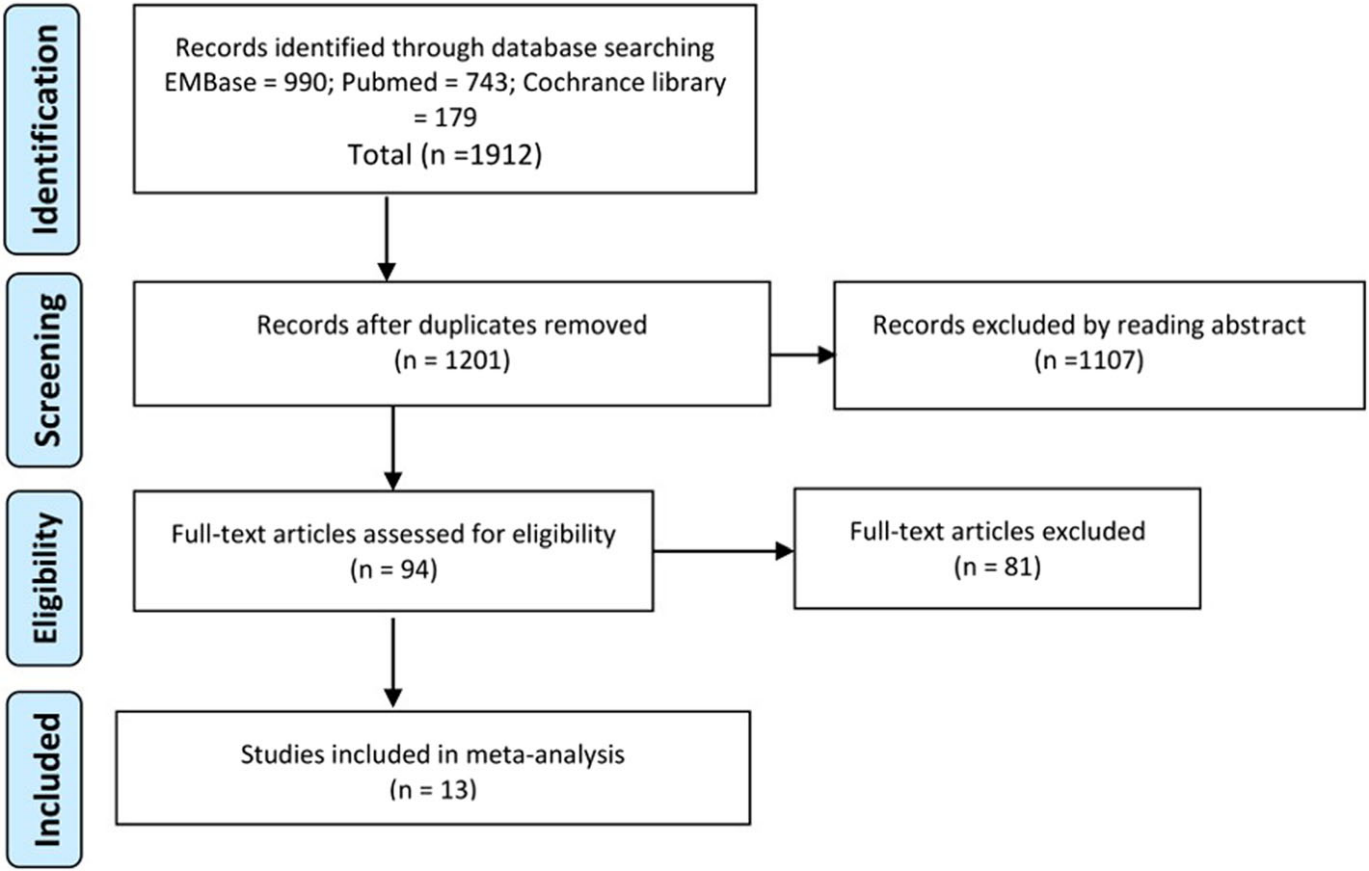
PRISMA flow chart

Figure 1 showed the flow chart of the literature search. The initial search found 1912 potentially relevant citations from PubMed (743), Embase (990), and the Cochrane Library (179). After the duplicates were removed, 1201 articles were included. After carefully screening the title and abstract, 94 citations were finally included removing the unrelated articles, case reports, systematic reviews, and non-comparative studies. The remaining 94 citations underwent full-text review, and 13 original reports meeting the inclusion criteria were selected.

### The characteristics of included studies

Overall, there were 13 studies (Table 1) [20–32] reporting on 1261 included TKAs. Both groups were well matched in age, BMI, and preoperative VAS, ROM, and knee deformity. NexGen Legacy Posterior Stabilized-Flex (PS-F) prostheses and NexGen Legacy Posterior Stabilized (PS) prostheses were used in the studies except one [25] in which the DePuy PFC Sigma fixed-bearing cruciate-substituting knee system was used in traditional MP approach total knee arthroplasties.

**Table 1:** Characteristics of included studies

Study/year	Country	Total TKAs		Follow-up (months)	Prosthesis type		Matching^a^
		QS	MP		QS	MP	
Huang 2016	China	31	30	65	PS-F	PS-F	1, 2, 3, 4, 5, 6, 7
Qi 2016	China	30	28	74.8	PS-F	PS-F	1, 2, 3, 4, 7
Chiang 2012	Taiwan	38	37	24	PS-F	PS-F	1, 2, 3, 4, 5, 6
Yang 2010	Korea	25	25	24	PS	PS	1, 2, 3, 4, 6
Karpman 2009	USA	20	19	6	CR	CR	1, 2, 3, 4, 6
Shen 2007	China	26	33	17	PS-F	PS-F	1, 2, 3, 4, 6, 7
Huang 2007	Taiwan	32	35	24	PS-F	PS-F	1, 2, 4, 6, 7
King 2007	USA	100	45	1.5	63PS/37PS-F	35CR/15PS	1, 2, 3, 4, 7
Kim 2007	Korea	120	120	21.5	PS	PS	1, 2, 3, 4, 6
Chin 2007	Singapore	30	30	Unclear	PS	Depuy CS	1, 2, 3, 4
Tashiro 2007	Japan	24	25	14	PS-F	23PS/2PS-F	1, 2, 3, 4, 5, 6, 7
Chen 2006	USA	32	38	33	PS-F	PS-F	1, 2, 3, 4, 6, 7
Kim 2006	Korea	144	144	13.6	PS	PS	1, 2, 3, 4

*QS* quadriceps-sparing, *MP* medial parapatellar, *PS-F* NexGen Legacy posterior stabilized-Flex prosthesis, *PS* NexGen Legacy posterior stabilized prosthesis, *CR* NexGen posterior cruciate-retaining prosthesis, *Depuy CS* Depuy PFC Sigma fixed-bearing cruciate-substituting prosthesis

^a^Matching: 1 sex, 2 age, 3 BMI, 4 follow-up, 5 preoperative VAS, 6 preoperative range of motion, 7 preoperative knee deformity

### Methodological quality assessment

The quality of RCTs was assessed by the tool recommended by the Cochrane Collaboration [17] which includes seven factors: random sequence generation, allocation concealment, blinding of participants, blinding of outcome assessment, incomplete outcome data, selective reporting, and other bias. The quality of retrospective studies was evaluated by modified NOS, which consists of three factors: patient selection, comparability of the study group, and assessment of outcome. Methodological quality of the included studies is shown in Table 2. RCTs achieving six or more scores assessed by the Cochrane risk of bias tool and retrospective studies achieving seven or more scores evaluated by the modified Newcastle-Ottawa Scale were considered to be of high quality.

**Table 2:** Quality assessment of included studies

Study/year	Study design	Tool	Quality score
Chiang 2012	RCT	^a^	7
Yang 2010	RCT	^a^	7
Karpman 2009	RCT	^a^	7
Kim 2007	RCT	^a^	4
Chin 2007	RCT	^a^	7
Huang 2016	Retrospective	^b^	7
Qi 2016	Retrospective	^b^	7
Shen 2007	Retrospective	^b^	8
Huang 2007	Retrospective	^b^	7
King 2007	Retrospective	^b^	8
Tashiro 2007	Retrospective	^b^	7
Chen 2006	Retrospective	^b^	7
Kim 2006	Retrospective	^b^	8

^a^Cochrane risk of bias tool

^b^Modified Newcastle-Ottawa Scale

### Results of meta-analysis

A radiographic outlier was defined as any knee alignment 4° or more outside of the ideal. A size of component which was 4 mm too small or too large and femoral notch greater than 2 mm was also considered as outliers [31]. And any component medialization or lateralization greater than 3 mm was considered outliers [30]. Meanwhile, one study defined the outliers as coronal tibial component angle 3° or more outside of the ideal, patellar tilt (> 5°), patellar subluxation (> 2 mm), and patellar resection asymmetry (> 3 mm) [28].

#### Outlier of hip-knee-ankle (HKA) angle

**Fig. 2:**
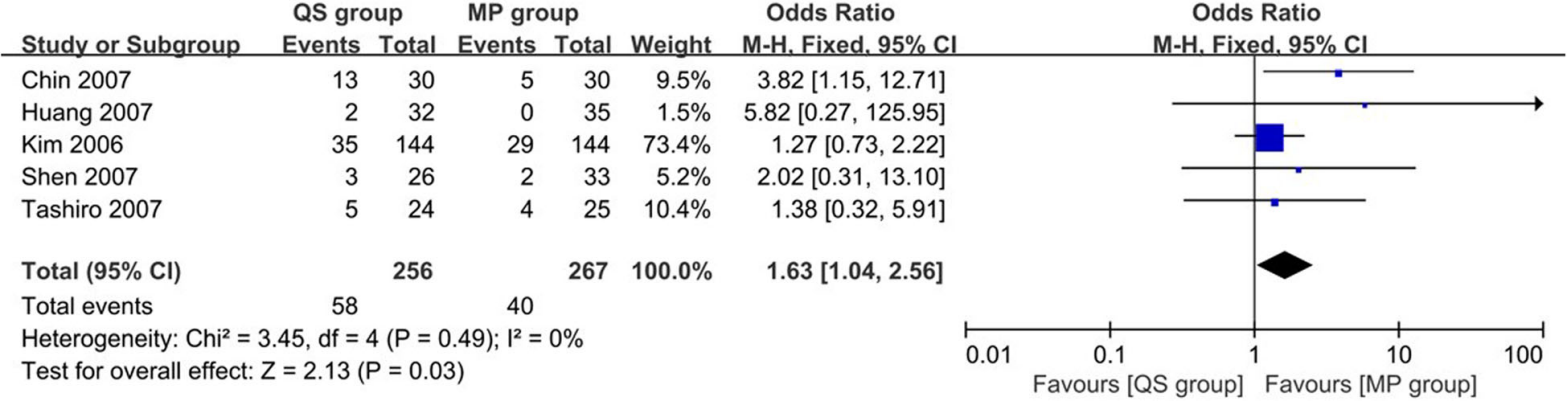
Forest plot of meta-analysis for the outlier of HKA angle

The outlier of HKA angle was reported in five studies [25, 26, 29, 30, 32] with 523 TKAs included. After pooling the whole data to process, we found that MP group had less outliers and the difference was not significant (OR 1.63, 95% CI 1.04 to 2.56, *p* = 0.03, Fig. 2). Meanwhile, there was no heterogeneity for the analysis of the outliers of HKA angle between studies (*I* ^2^ = 0%, *p* = 0.49).

#### Outlier of coronal femoral component angle

**Fig. 3:**
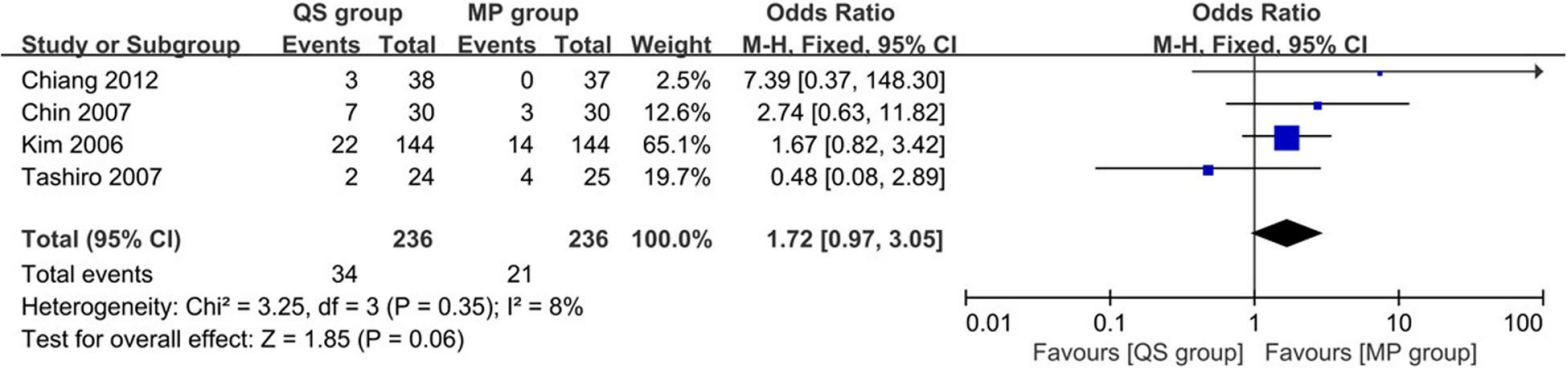
Forest plot of meta-analysis for the outlier of coronal femoral component angle

Pooling the data of four studies [22, 25, 30, 32] included 472 TKAs that reported MP group got better femoral component position, but the difference was not statistically significant (OR 1.72, 95% CI 0.97 to 3.05, *p* = 0.06, Fig. 3) and the heterogeneity was low (*I* ^2^ = 8%, *p* = 0.35).

#### Outlier of coronal tibial component angle

**Fig. 4:**
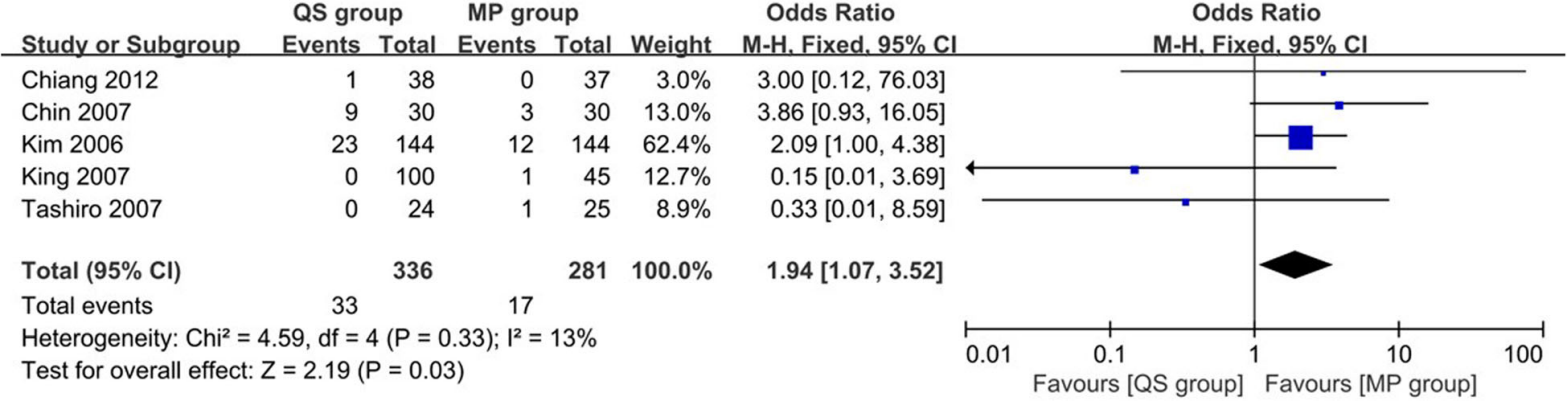
Forest plot of meta-analysis for the outlier of coronal tibial component angle

Five studies [22, 25, 28, 30, 32] reported the outlier of coronal tibial component angle in 617 TKAs, and the data showed a significant difference favoring the MP group (OR 1.94, 95% CI 1.07 to 3.52, *p* = 0.03, Fig. 4) with low heterogeneity (*I* ^2^ = 13%, *p* = 0.33).

#### Outlier of femoral notch

**Fig. 5:**
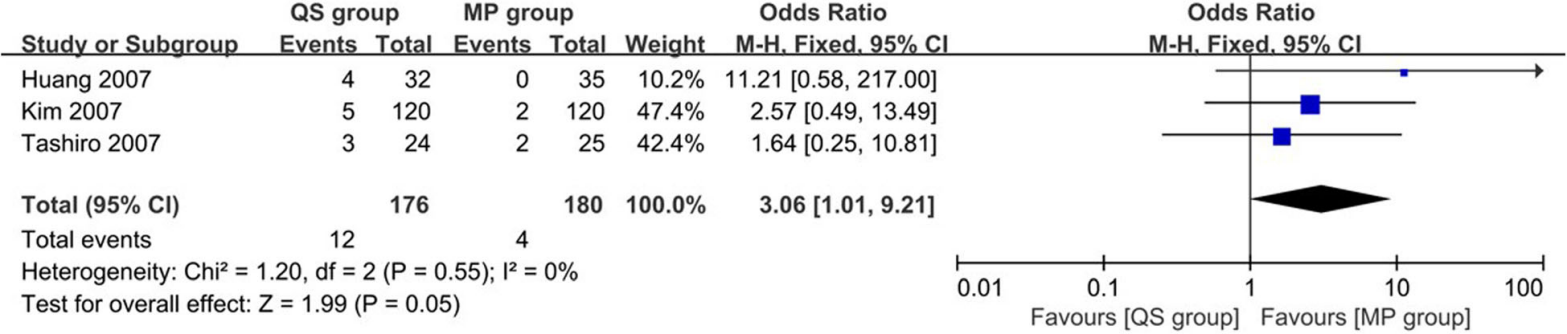
Forest plot of meta-analysis for the outlier of femoral notch

Three studies [26, 27, 30] including 356 TKAs evaluated the outlier of femoral notch. Pooling data showed a lower incidence of outlier in the MP group than in the QS group (OR 3.06, 95% CI 1.01 to 9.21, *p* = 0.05, Fig. 5) with no heterogeneity (*I* ^2^ = 0%, *p* = 0.55).

### Publication bias

**Fig. 6:**
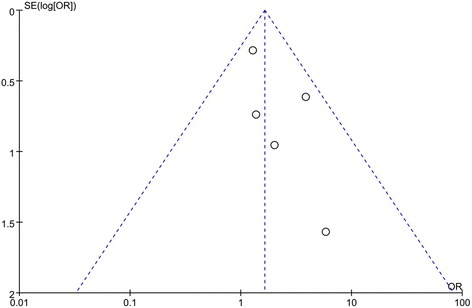
Funnel plot illustrating meta-analysis of the outlier of HKA angle

Figure 6 shows a funnel plot of the studies included in this meta-analysis that reported the outlier of HKA angle. All studies lie inside the 95% CI, with an even distribution around the vertical, indicating no obvious publication bias.

## Discussion

The major finding of this meta-analysis was that the traditional MP approach was superior to the QS approach in outliers of HKA angle, coronal tibial component angle, and femoral notch. There were no statistical differences between the two groups in the outlier of coronal femoral component angle.

However, seven studies [24, 25, 27, 29–32] demonstrated that there was no significance between the two groups concerning hip-knee-ankle (HKA) angle. And more researches showed that there was no difference between the two groups relating to coronal femoral component angle and coronal tibial component angle [21, 23, 25, 27, 30–32], except Chiang et al. [22] reporting the MP approach acquiring more ideal angle in coronal femoral component angle and two [22, 23] in coronal tibial component angle. With regard to the sagittal component angle, some studies [21, 23, 25, 27, 30–32] showed that there was no difference between the two groups in sagittal femoral component angle and some studies [21, 24, 25, 27, 30–32] demonstrated the same results about the sagittal tibial component angle except only one [23] indicating more accurate outcome. Three studies [23, 27, 28] showed that the same patellar tilt could be gained by any technique and one study [28] showed that there was no significant difference of patellar dislocation and patellar resection asymmetry between the two groups. But Huang et al. [20] considered that QS approach could achieve better patellar tilt and lateral patellar dislocation than MP approach in the long-term follow-up due to not everting the patellar during QS approach surgery. Furthermore, this study showed that patellar alignment had no correlation with the clinical scores, such as knee society score (KSS), Western Ontario and McMaster University Osteoarthritis Index (WOMAC), and the Short Form 36 (SF-36). But some studies [24, 31, 32] showed that the difference of the outliers of component size was not significant between the two groups.

King et al. [28] showed the learning curve of the QS approach in TKA for high-volume arthroplasty surgeons who could achieve the comparable limb alignment and component position between the two groups after completing just over 25 QS procedures and optimized patellar alignment after about 50 procedures.

Overall, due to the limited visualization of anatomic landmarks and the side-cutting instruments, the QS approach tends to have the most outliers, and MP approach with patellar eversion tends to provide good visualization for aligning the components accurately. However, the prolonged rehabilitation and postoperative pain of MP approach promote the development of minimally invasive surgeries, and experienced surgeons out of the learning curve could achieve the same good radiological outcomes using both MP and QS approaches [5, 23, 24, 28]. So workshop in cadavers and the number of cases and regular surgeries are important. In order to avoid malalignment and malposition, surgeons could increase quadriceps exposure centimeter by centimeter from the upper pole of the patellar to make sure the accuracy of the osteotomy. Besides, in view of the smaller injury to the quadriceps to get better earlier clinical outcomes, we should improve instruments and techniques that will strengthen the role of QS in TKA with significantly reduced number of outliers in the learning curve.

This is the first meta-analysis and systematic reviews that directly compared the radiological outcomes for QS approach and traditional MP approach in TKA. But the following limitations of this meta-analysis must be taken into account. The main limitation is that all the included studies were retrospective, except for five RCTs with one achieving very low quality score. Inadequate random sequence generation and blinding tended to increase the risk of bias. In addition, the included studies were carried out by different levels of surgical expertise and some studies were in the learning curve. Furthermore, the number of included studies and the sample size analyzing every outcome are relatively small. Future large-volume, well-designed RCTs with comprehensive measurements are waited to confirm and update the findings of this meta-analysis.

## Conclusion

In conclusion, MP approach in TKA gives superior limb alignment, component position, and the accuracy of the osteotomy, especially in the outlier of HKA angle, coronal tibial component angle, and femoral notch.

## Abbreviations

BMI: Body mass index
CI: Confidence interval
CR: NexGen posterior cruciate-retaining prosthesis
Depuy CS: Depuy PFC Sigma fixed-bearing cruciate-substituting prosthesis
HKA: Hip-knee-ankle
KSS: Knee society score
MP: Medial parapatellar
MV: Midvastus
NOS: Newcastle-Ottawa Scale
OR: Odds ratio
PS: Posterior stabilized
PS-F: Posterior stabilized-flex
QS: Quadriceps-sparing
RCT: Randomized controlled trial
ROM: Range of motion
SF-36: Short Form 36
SV: Subvastus
TKA: Total knee arthroplasty
VAS: Visual analog scale
WOMAC: Western Ontario and McMaster University Osteoarthritis Index
